# Investigation of the diagnostic importance and accuracy of CT in the chest compared to the RT-PCR test for suspected COVID-19 patients in Jordan

**DOI:** 10.12688/f1000research.130388.1

**Published:** 2023-06-26

**Authors:** Haytham Alewaidat, Ziad Bataineh, Mohammad Bani-Ahmad, Manar Alali, Ali Almakhadmeh

**Affiliations:** 1 Applied Medical Sciences, Jordan University of Science and Technology, irbid, 22110, Jordan; 2 Anatomy, Jordan University of Science and Technology, Irbid, 22110, Jordan; 3 Medical Laboratory Science, Jordan University of Science and Technology, Irbid, 22110, Jordan; 4 Medical Laboratory Science, Zarqa University, Zarqa, Jordan; 5 Radiologic Technology, Jordan University of Science and Technology, Irbid, 22110, Jordan

**Keywords:** COVID-19, X-ray, epidemics, Radiographer, knowledge

## Abstract

**Background**: COVID-19 affects different people in different ways. The illness varies from mild to acute. Mild illness is treated even without hospitalization. RT-PCR is one of the main techniques, which are used to diagnose COVID-19, but in this paper, we have investigated that Chest CT is a more efficient alternative option to RT-Polymerase Chain Reaction. The purpose of our study is to diagnose the importance of chest CT in comparison to the RT-PCR test method for the patients who might have COVID-19 virus. The study will aid in contrasting the performance of chest CT method and RT-PCR method.

**Methods:** This retrospective study included 1276 patients of the Jordanian hospitals' medical database that reception and following of suspected Covid-19 patients receiving high-resolution chest CT and real-time RT-PCR. Patients chosen underwent both chest CT and RT-PCR examinations, and the performance of chest CT in the diagnosis of COVID-19 evaluated, with maintaining the RT-PCR test as the reference standard.

**Results:** The sensitivity and accuracy of chest CT in identifying COVID-19 were all higher in patients over 60 than in those under 60, with no difference in positive predictive values and negative predictive values. The accuracy in-patient under 60 is higher than over 60 patients. Males had a higher specificity of chest CT in the diagnosis of the COVID-19 virus than females, but there was no difference in sensitivity, negative predictive value, positive predictive value, or accuracy.

**Conclusions: **RT-PCR is considered as the great standard for the diagnosis of Covid-19. According to the findings of our study, the best alternative option to RT-PCR is chest CT scan. CT scan is the less sensitive method but faster than RT-PCR. In a clinical setting, a radiologist with adequate training can distinguish the virus COVID-19 from other viral-induced pneumonias.

## Introduction

As the novel COVID-19 virus has been spreading, a vast range of knowledge has been gained on it. A series of new studies provide guidance about the use of RT-PCR test is usually adopted for the COVID-19 diagnosis and the use of chest CT diagnostics.

The COVID-19 virus is a recent extremely infectious disease that has infected the entire globe. This virus belongs to coronaviridae family of RNA-surfaced viruses which are actually widely found in humans but also in the mammals as well as birds, also responsible for infecting the epithelial cells of human airways that cause serious and even fatal respiratory diseases, particularly in older patients with comorbidity such as hypertension and also the patients with diabetes mellitus have a greater chance to have a more severe progression of the illness and have also an elevated rate of ICU admission death from COVID-19 disease. So, all the necessary measure should be taken by the patients with comorbidities to avoid becoming infected with SARS CoV-2, as their prognosis is usually the worst. ^1^ Diagnoses rely on three cardinal major clinical findings, including fever as well as cough and breathe shortness. The test which is being done using RT-PCR was regarded a very good quality for the detection of viral COVID-19 that performed on the nasopharyngeal and also with the oropharyngeal swabs, sputum, the blood samples, the body fluids as well as stool sample, and Broncho-alveolar lavage fluid, ^2^^,^^3^ but RT-PCR detection sensitivity in real-time has been shown to be lower than that for chest CT. Despite to negative RT-PCR test, clinically suspect COVID-19 pneumonia in patients may be found in CT chest. ^4^ The problem is additionally complicated by the fact that usually a longer time period (several hours) is required for RT-PCR results and in the very initial stages of an infectious disease a fast decision-making is needed, while the CT scan results of suspected patients can be obtained in a matter of minutes. ^5^

Many studies show different levels of RT-PCR sensitivity, ranging in range from 37 percent to 71 percent during Wuhan outbreak, ^6^^,^^7^ this is presumably due to immature production of nucleic acid detection technology and the level of viral RNA being below the control of detection of the test, difference in the detection rate of different manufacturers and sample type, and also the low load of virus in patient, sample acquisition time, or insufficient sampling in clinic. ^8^^,^^9^ Furthermore, the person skill to acquire the sample and the time period between symptom manifestation and testing influence the results of the RT-PCR. The shortcomings of RT-PCR have prompted some studies to propose that a CT scan be performed. ^4^^,^^7^

During the detection of Covid-19, management and safety of patients suspected with Covid-19, an important role is being played by medical imaging in promoting the clinical aspect of decision-making, ^10^ the primary imaging modality for investigating suspicious COVID patients is the chest X-ray, ^11^ CT is not well known as standard means for suspected patients with COVID-19, but sometimes CT, rather than chest x-rays, is identified for certain problems related to the mechanical ventilation (pneumonia, pneumothorax, and emphysema). ^3^ Chest CT appears to be accurate, available, and realistic especially in areas of high incidence and prevalence, ^12^^,^^13^ it gives us a strong non-invasive exam. ^14^ The high sensitivity of CT (88-97 percent) was confirmed by Ai et al., ^6^ but low specificity was reported (as low as 25 percent), with actually an validity of 68 percent for the detection of COVID-19, so the backing for detecting COVID-19 virus is RT-PCR and negative CT imaging does not exclude infection with COVID-19, but CT partially overcomes the limitation of RT-PCR. ^15^

An important role is being played by chest CT for diagnosing the patients who already have a covid-19 symptom and high suspicion, ^16^ in addition to being able to manage covid-19 patients, ^17^ it is useful in asymptomatic patients for the identification of the disease. ^18^^,^^19^ Shuchang zhou, ^20^ defining the CT characteristics of this epidemic disease in Wuhan, CT analysis showed that the disease has a mixture and a diversity of patterns. The common features related to COVID-19 cases that are being detected are the ground-glass opacities (GGO) which was present at the early stage and consolidative opacity, ^14^^,^^21^ pleural effusion in the advanced phase can occur. The predominant distribution of multifocality and lesions was a very characteristic manifestation in the middle as well as lower zone and posterior region of the lung.

Since the RT-PCR has several limits, there is a restriction on chest CT scan for COVID detection as well. The outcomes of CT depend on the expertise of the radiologists who diagnose the suspected patients, ^22^ as well as the need to sterilize the device for suspected patients after the time of use. If they do not take this into account, the device may be more of a source of infection than people. Chest CT cannot, due to these restrictions, be used as an independent diagnostic method to exclude or confirm COVID-19. The diagnostic standard and the main factor in the decision-making process are the RT-PCR test results.

In March 2020, the thoracic radiology society and the U.S. Emergency Radiology Society stated that “at this time, do not recommend routine CT screening for the diagnosis of patients under investigation for COVID-19”. ^23^ In order to determine whether CT scans could be used to diagnose COVID-19, the current retrospective study was therefore designed to collect all existing data related to the COVID-19 detection validity of chest CT scans. An attempt to establish the link among the results of CT scan and results of positive RT-PCR by contrasting the CT imaging sensitivity and testing of RT-PCR at the presentation and to show whether the CT scan is more effective in diagnosing suspicious patients with RT-PCR negative result is also made.

The purpose of our study is to diagnose the importance of chest CT in comparison to the RT-PCR test method for the patients who might have COVID-19 virus. The outcome objectives will give us the idea of how to provide the target population the knowledge gained as a result of the study. Objectives could be either for short term or for long term. The study will aid in contrasting the performance of chest CT method and RT-PCR method. It will help us in providing the results of whether chest CT test is more effective than the RT-PCR for COVID-19 detection in suspected patients or not. If RT-PCR gives us the false positive or false negative results is one of the main important answers to question that we will get through the outcome results of the study. Patients’ clinical history will aid in providing the information whether the patients with already having some kind of disease are more susceptible to COVID-19 or not. The other learning outcome will be if the accuracy in patients under 60 is higher than over 60 patients or not. Also, if the males have an elevated precision in Covid-19 detection than females, is also one main point that is going to be discussed in the paper.

## Methods

### Study design and participants

This retrospective study involved total 1276 patients of the Jordanian hospitals’ medical database that reception and following of suspected patients having Covid-19 receiving the chest CT of high-resolution and RT-PCR. The study was approved by JUST institutional review boards (IRB) (2020865) approved it; signed informed consent was waived since the study is of a retrospective design. Patients chosen had to go through the chest CT as well as RT-PCR. In the diagnosis of COVID-19 suspected patients, the evaluation of chest CT scan was done, with maintaining the RT-PCR test used as the standard reference.

Details of patients, for example, gender, age (≥18 years), date and timing of the RT-PCR test, date and timing of the chest CT test, No. of tests performed and the time between consecutive tests (d), effective transformation of RT-PCR test results (negative to positive and vice versa), chest CT findings, available clinical history, and symptoms are recorded. An (ID) was assigned to each enrolled patient, which was then used to gather the patient’s personal information. This protocol guaranteed anonymity and sensitive data not to be revealed.

### Data sources of RT-PCR results

Data were collected from control center databases to symptomatic patients who undergoing COVID-19 laboratory tests with initial positive results and negative results of RT-PCR.

### Chest CT protocol

Supine position was being used to take images of COVID-19 suspected patients. High Resolution Chest CT, the used parameters for the scanning were: tube voltage, 120 kVp; automatic tube current modulation; tube current, 30-70 mAs; pitch, 0.99-1.22 mm; matrix, 512 × 512; slice thickness, 10 mm, and field of view, 350 mm × 350 mm.

### Image analysis

The CT image was analyzed separately, inconsistencies were resolved by consensus between two thoracic radiologists blinded to clinical evidence, but epidemiological history and clinical symptoms were available. The reporting of whether the image features (ground-glass opacities (GGO), consolidation, GGO and consolidation, bronchiectasis of traction, thickening of the bronchial wall, reticulation, sub-pleural bands, vascular enlargement, distribution of lesions, plural effusion, crazy paving, and reserved halo) were present or not was being done. The shortest time period among the RT-PCR test and the chest scan (≤7 d) was selected in the COVID-19 suspected patients with the number of CT tests. If the time in the middle of chest CT and RT-PCR tests was more than a week, patients were kept out. The patient whose test result confirmed is negative (RT-PCR and chest CT) take as a case-control.

### Statistical analysis

The reverse transcriptase (RT) enzyme is used to convert viral RNA into DNA, which is subsequently analyzed by the polymerase chain reaction (PCR). For one thing, it has a great degree of precision. False-positive findings are exceedingly rare, as are negative test results, which are virtually always true. Low sensitivity is a concern for this test, as reported by Schafer-Prokop in the literature. In addition to the length of symptoms, quality of the sample, and assay employed, there are a number of other factors that might determine how sensitive a test is. CT sensitivity, on the other hand, is deemed to be above 90%. ^24^^,^^25^ In other words, the infection will be discovered in at least 90 out of every 100 affected individuals. That’s a wonderful idea, but it might be an issue if the sickness isn’t widely distributed. “With prevalence less than 10%, CT is not ideal for screening or primary diagnosis, as perhaps there will be too many false positives,” he says. During the winter months, it is especially crucial to keep an eye out for similar CT results from other viral illnesses.

PRISMA criteria were used while running an exploratory systematic review and meta-analysis. Studies involving the comparison of the diagnostic abilities of “Chest CT” scans with (“RT-PCR”) were sought by using computerized databases. Sensitivity, specificity, and accuracy were the three most critical outcome markers to focus on. By comparison, “RT-PCR” was shown to be 0.91 (0.82-0.98) sensitive, 0.75 (0.25-0.001) specific, and 0.87 (0.68-0.99) accurate when used as the reference. “RT-PCR” had a statistically significant advantage over “Chest CT” in terms of accuracy. In this study, the P-value was 0.001 and the Odds Ratio [OR] was 0.22. “Chest CT” does not have the same level of specificity as “RT-PCR”. Opacities and consolidations in the ground-glass were the most prevalent “Chest CT” symptoms. Early studies tended to favor “Chest CT” over future bigger investigations; a tendency that remained persistent. In terms of recognizing COVID-19, “Chest CT” is less accurate than “RT-PCR.” If a patient’s symptoms are suspicious but an “RT-PCR” test fails to detect SARS-CoV-CoV-2 or COVID-19, the test may still be beneficial in verifying the presence of the virus.

## Result

**Figure 1.**
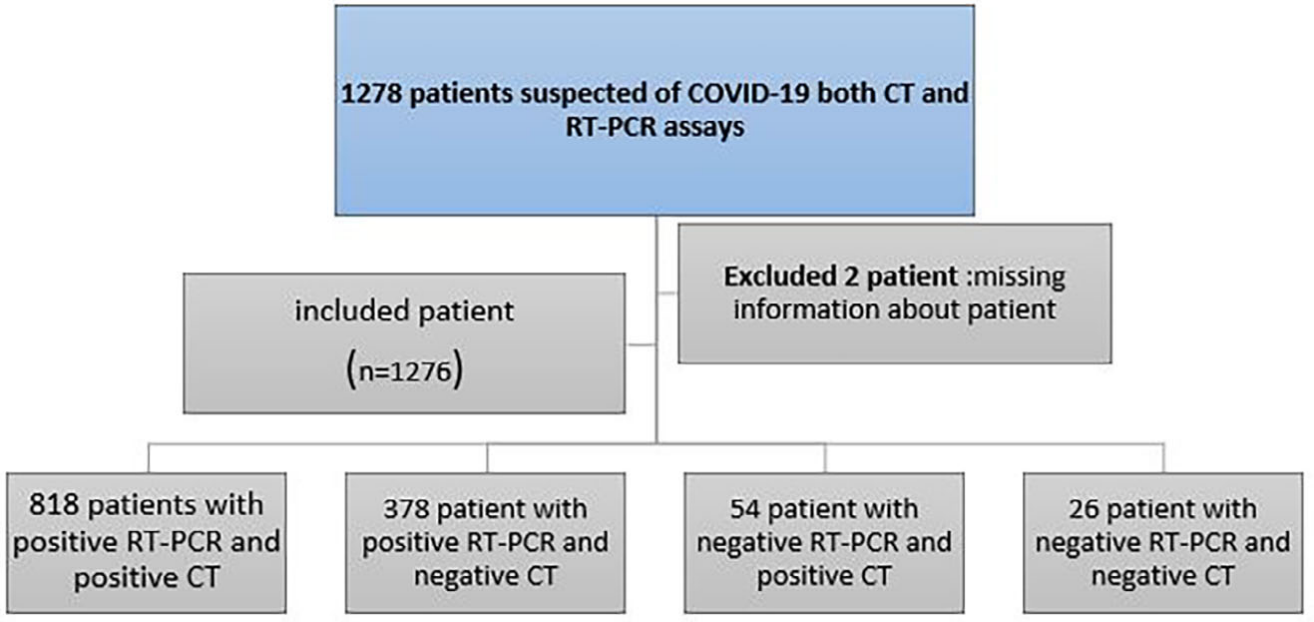
Study flowchart. COVID-19 = coronavirus 2019, RT-PCR = reverse-transcription polymerase chain reaction.

As a result of missing data, two patients were excluded. After these patients were excluded, 1276 patients (756 men [59.25%], 520 females [40.75%]) were available for analysis. The study flowchart is depicted in Figure 1.

There were 1196 initial positive results of RT-PCR test for COVID-19 among the 1276 patients, and 80 initial negative for RT-PCR test results, for actually a positive rate of 93.73% ( Figure 1). There were 818 positive results of chest CT scans among the 1196 patients who had initial positive RT-PCR results. 54 of the 80 patients who had an initial negative result for RT-PCR test also had a positive chest CT scan.

Twenty patients change their initial result from positive to negative, 66 patients change from negative to positive, 104 patients repeat the test and still positive. After this change, the positive RT-PCR be 1242 and the negative be 34, 1086 of patients was the result positive from the initial test, 150 patients take two exams to detect the virus, 30 patients take three exam and 10 patients take four exams to detect the positive result. 92 patients have 0-3 days’ time between consecutive tests, 98 has ≥4 days ( Table 1).

**Table 1.** Details of multiple RT-PCR assays in 1276.

Characteristic	Patient
No. of tests performed	
2	150
3	30
4	10
Time between consecutive test (d)	
0-3	92
≥4	98
Dynamic change	
From positive to negative result	20
From negative to positive result	66
Same positive result	104
No repeat test	1086

Between the two, the median time of paired chest CT exams and RT-PCR laboratory tests was actually one day (range, 0–7 days). 872 (68.34%) of the 1276 patients had positive result for chest CT findings. Ground-glass opacity (574 of 1276 patients [45%]) and consolidations (413 of 1276 patients [32.4%]) were the most common chest CT findings ( Table 2), (404 of 1267 patients [31.66%]) with no CT findings.

**Table 2.** Performance of chest CT in the diagnosis of COVID-19.

Summary of patient characteristics		
Characteristics		Value
No. of patient		1276
Age		
18-39		160
40-59		416
60<		700
No. of male		756
No. of female		520
Median time between chest CT and RT-PCR assay (d)		1 d *
Result of RT-PCR assay		
Positive		1242
Negative		34
Finding and manifestations of chest CT		
Consistent with viral COVID-19 (positive)		872
Ground-glass opacity		574
Consolidation		413
Mixed GGO and consolidation		121
Traction bronchiectasis		52
Bronchial wall thickening		54
Reticulation		34
Sub-pleural bands		26
Vascular enlargement		10
Lesion distribution		2
Plural effusion		52
Crazy paving		37
Reserved halo		16
No CT findings		404

* day.

Regarding the patient’s clinical history, 688 positive PCR patients have hypertension [57.5%], 572 have diabetes [47.8%], 56 have chronic respiratory disease [4.7%], 293 have cardiovascular disease [24.5%], 121 have chronic kidney disease, 53 have cancer [4.4%], and 120 smoke [10%].

The majority of the patients had (fever 696 [54.5%], cough 797 [62.4%], and SOB 828 [64.9%]). Patients experienced muscle soreness 282 [22.1%], fatigue 318 [24.9%], sore throat 136 [10.7%], headache 174 [13.6%], sputum production 146 [11.4%], chest pain 218 [17.1%], chills or/and rigors 298 [23.4%], diarrhea 128 [10%], and appetite 180 [14.1%]. Dizziness 40 [3.1%], loss of taste and/or smell 42 [3.3%], vomiting 96 [7.5%], abdominal pain 74 [5.8%], nausea 84 [4.4%], weakness 10 [8%], LOC 16 [1.3%], and drowsiness 4 [0.3%] are the least common symptoms. Twenty patients (1.6%) are asymptomatic.

In 190 people, chest CT imaging showed positive results for COVID-19, although its initial RT-PCR results from samples taken from nasopharyngeal swab were incorrect. Results of 66 patients shifted from negative to positive, 20 patients’ results changed from positive to negative, and 104 patients’ results did not change ( Table 3). There were total 872 patients who have the positive results for chest CT findings (<60 years, n = 330; ≥60 years, n = 542; 530 men, 342 women). The sensitivity, specificity, and accuracy of chest CT in indicating COVID-19 infection were 68.39% (95% CI: 66%, 71%; 818 of 1196 patients), 32.5% (95% CI: 22%, 44%; 26 of 80 patients), and 66.14% (95% CI: 63%, 69%; 844 of 1276 patients), respectively, with RT-PCR results taken as the standard of reference ( Table 4). In Table 2, the performance of chest CT in diagnosing COVID-19 in the age and sex groups, which differ from each other, were reported. The sensitivity and accuracy of chest CT in identifying COVID-19 were all higher in patients over 60 than in those under 60, with no difference in positive predictive values and negative predictive values. The accuracy in-patient under 60 is higher than over 60 patients. Males had a higher specificity of chest CT in the diagnosis of the COVID-19 virus than females, but there was no difference in sensitivity, negative predictive value, positive predictive value, or accuracy.

**Table 3.** Patient general characteristics.

**#No at all…**		
No at all: 1276		
All initial pos pcr: 1196	All pos CT: 872	
All end pos pcr: 1242		
All initial neg pcr: 80	All neg CT: 404	
All end neg pcr: 34		
**According to gender:**		
All male: 756	All female: 520	
All initial pos male: 696	All pos female: 500	
All initial neg male: 60	All neg female: 20	
All Male pos CT: 530	All female neg CT: 178	
All Male neg CT: 226	All female pos CT: 342	
**According to age:**		
(18-39)	(40-59)	(Above 60)
All (18-39): 160	All (40-59): 416	All (Above 60): 700
All pos pcr (18-39): 148	All pos pcr (40-59): 382	All pos pcr (Above 60): 666
All neg pcr (18-39): 12	All neg pcr (40-59): 34	All neg pcr (Above 60): 34
All pos CT (18-39): 78	All pos CT (40-59): 252	All pos CT (Above 60): 542
All neg CT (18-39): 82	All neg CT (40-59): 164	All neg CT (Above 60): 158
**According to test interval:**		
All (0-3) test	92	
All (≥4) test	98	

**Table 4.** Performance of chest CT in the diagnosis of COVID-19.

Result					Test performance				
Parameter	TP	TN	FP	FN	Sensitivity	Specificity%	PPV (%)	NPV (%)	Accuracy (%)
Overall	818	26	54	378	68.39 (818/1196) [66,71]	32.50 (26/80) [22,44]	94 (818/872) [93,95]	6.44% (26/404) [5,9]	66.14 (844/1276) [63,69]
Age									
<60 y	306	22	24	224	57.74 (306/530) [53,62]	47.83 (22/46) [33,63]	92.73 (306/330) [90,94]	8.94 (22/246) [7,12]	56.94 (328/576) [53,61]
≥60 y	512	4	30	154	76.88 (512/666) [73,80]	11.76 (4/34) [3,27]	94.46 (512/542) [94,95]	2.53 (4/158) [1,6]	73.71 (516/700) [70,77]
Sex									
M	486	16	44	210	69.83 (486/696) [66,73]	26.67 (16/60) [16,40]	91.7 (486/530) [90,93]	7.08 (16/226) [5,11]	66.4 (502/756) [63,70]
F	332	10	10	168	66.4 (332/500) [62,70]	50 (10/20) [27,73]	97 (332/342) [95,98]	5.62 (10/178) [4,8]	65.77 (342/520) [61,70]

## Discussion

The understanding of COVID-19 diagnosis and treatment approaches is a fast-expanding environment, with new knowledge regarding the infection being gained on a weekly basis. Preliminary investigations are needed to further understand CT scans’ ability to identify COVID-19 in individuals with symptoms and in the first stages of infection. CT scans can have a considerable impact on patients suffering from COVID-19 indications who do have a need of rapid therapy, and this is especially true in symptomatic patients. For symptomatic comparisons, CT scans may be more sensitive than conventional RT-PCR, but their value in asymptomatic persons, is still being contested, according to existing information. CT imaging can be critical in the evaluation of COVID-19 in both symptomatic and asymptomatic persons since it is usually available in almost every healthcare facility worldwide and the results are rapidly available.

The chest CT scan is a noninvasive imaging procedure that is both accurate and quick. Architectural distortion in peripheral distribution and multifocal organizing pneumonia are the varying degrees of characteristic CT features in the disease process that can be found in the Covid-19 patients as per the findings published in recent literature. ^26^ The RT-PCR is a technique used as a great standard for the diagnosis of virus COVID-19, although it can produce false-negative results in initial stages of disease in some or many patients. In a number of individuals who had a false-negative RT-PCR result, CT scans validated the diagnosis. ^27^ RT-PCR can also give false positive results. No doubt, there is too limited information about false positives but it has been identified that false positives will depend on the length of DNA probes and how many and which genes are used to get measured in the RT-PCR. Some technical errors could also be responsible for the false results. The main reasons of the false positive results are actually the laboratory errors. Contamination or testing the wrong sample could be responsible for false positive results. False positive results are the results in which the suspected patient does not have the COVID-19 but the results show that the patient have the virus. Chest CT may be regarded a major technique for detecting current COVID-19 in epidemic locations in these instances. We discovered changes in CT characteristics among the groups with negative and positive initial RT-PCR results in this investigation.

Sensitivity of 96% and specificity of 62% yield is shown by initial RT-PCR in one of the studies, which is actually little higher than the previous reports. The accuracy of RT-PCR can be influenced by different elements which includes load of the virus in respiratory tract, source of sample, procedures of sample and the authenticity of testing kits. ^28^ As a result, RT-PCR test findings should be regarded with caution. Furthermore, according to our findings, almost every patient had shown positive result with chest CT scan right before and within 6 days of initial PCR results that were positive. In this study, about 68 percent of patients (872 of 1276) had classic CT attributes accordant with COVID-19 before the initial positive RT-PCR results, which was lower than the estimate reported by Guan et al (86.2 percent). ^25^ This suggests that computed tomography (CT) could be beneficial in detecting suspected instances early on.

The sensitivity, specificity, and accuracy of chest CT in detecting COVID-19 infection were 68.39% (818 of 1196 patients), 32.5% (26 of 80 patients), and 66.14% (844 of 1276 patients), respectively, using RT-PCR data as the reference standard in 1267 patients. The positive predictive value was 94% (818 of 872 patients) and the negative predictive value was 6.44% (26 of 404 patients), respectively. This demonstrate that the sensitivity and accuracy of CT were moderate, but the specificity was poor because of COVID-19’s CT appearance is similar to that of other viral pneumonias such as influenza, parainfluenza, adenovirus, respiratory syncytial virus, rhinovirus, and human metapneumovirus. ^29^ Despite concerns about specificity and sensitivity, the RT-PCR test has been regarded as one of the great standards for the diagnosis of COVID-19. In the monitoring of disease spread, many factors play a significant role. The RT-PCR test is actually time consuming and also the availability of kits is one of the main limiting factors. COVID-19 chest CT manifestations are being identified, but a large amount of findings are not available regarding its course and treatment till date. Although, X-ray is the first way of diagnosis for COVID-19, hence, CT is the better alternative in the recognition of disease and check-up. For patient surveillance, an X-ray examination can be done in advance of RT-PCR test results.

The current investigation was carried out by evaluating the medical records of COVID-19 patients, the majority of whom had a clear contact. We discovered that general and respiratory symptoms are frequent in COVID-19 patients, such as (fever, cough, and SOB). According to the present data, a single indication or symptom is insufficient to rule in or rule out COVID19. On the other hand, some combinations of indications and symptoms could be proven useful in case of prioritizing people for more testing. ^24^ The present article discusses the comorbidities related to COVID-19 disease, which include hypertension, heart disease, diabetes, respiratory disease kidney disease, malignancy, and smoking. It is equally important how quickly we manage to diagnose the disorder which is induced by the virus in a person with certain comorbidity. This will allow us to provide the suitable treatment plan for the person in a shielded timeframe.

Our study had several limitations, including the difficulty of obtaining patients diagnosed with RT-PCR and computed tomography at the same time due to our reliance on PCR examination as a better solution and reference diagnosis, which was evident in the CT scan related sensitivity and accuracy, and the need to improve this and focus more on this quick alternative scan. Second, there was limited clinical and laboratory data during the critical period of COVID-19, because all the regional hospitals were already filled.

## Conclusion

RT-PCR is considered as the great standard for the diagnosis of Covid-19. According to the findings of our study, the best alternative option to RT-PCR is chest CT scan. CT scan is the less sensitive method but faster than RT-PCR. In a clinical setting, a radiologist with adequate training can distinguish the virus COVID-19 from other viral-induced pneumonias. In RR-PCR positive COVID-19 cases, chest CT is found which is normal, and if we talk about RT-PCR negative cases, typical CT manifestations can be found. Chest CT should be used to reduce the possible risk of giving false-negative results for suspected COVID-19 patients.

## Data Availability

Zenodo: Investigation of the diagnostic importance and accuracy of CT in the chest compared to the RT-PCR test for suspected COVID-19 patients in Jordan, https://doi.org/10.5281/zenodo.7684523. ^30^ Data are available under the terms of the Creative Commons Attribution 4.0 International license (CC-BY 4.0).

## References

[1] Chen Z-H : Chest CT of COVID-19 in patients with a negative first RT-PCR test: Comparison with patients with a positive first RT-PCR test. *Medicine.* 2020;99(26):e20837. 10.1097/MD.0000000000020837 32590775 PMC7328934

[2] Corman VM : Detection of 2019 novel coronavirus (2019-nCoV) by real-time RT-PCR. *Eurosurveillance.* 2020;25(3). 10.2807/1560-7917.ES.2020.25.3.2000045 31992387 PMC6988269

[3] Karam M : Chest CT versus RT-PCR for the Detection of COVID-19. 10.1177/20542704211011837 PMC8127597 34035931

[4] Long C : Diagnosis of the Coronavirus disease (COVID-19): rRT-PCR or CT? *Eur. J. Radiol.* 2020;126:108961. 10.1016/j.ejrad.2020.108961 32229322 PMC7102545

[5] Jawerth N : How is the COVID-19 virus detected using real time RT-PCR. *IAEA Bull.* 2020;8–11.

[6] Ai T : Correlation of chest CT and RT-PCR testing in coronavirus disease 2019 (COVID-19) in China: a report of 1014 cases. *Radiology.* 2020;200642. 32101510 10.1148/radiol.2020200642 PMC7233399

[7] Li Y : Stability issues of RT-PCR testing of SARS-CoV-2 for hospitalized patients clinically diagnosed with COVID-19. *J. Med. Virol.* 2020. 10.1002/jmv.25786 PMC7228231 32219885

[8] Falaschi Z : Chest CT accuracy in diagnosing COVID-19 during the peak of the Italian epidemic: A retrospective correlation with RT-PCR testing and analysis of discordant cases. *Eur. J. Radiol.* 2020;130:109192. 10.1016/j.ejrad.2020.109192 32738464 PMC7382359

[9] Zou L : SARS-CoV-2 viral load in upper respiratory specimens of infected patients. *N. Engl. J. Med.* 2020;382(12):1177–1179. 10.1056/NEJMc2001737 32074444 PMC7121626

[10] Pan Y Guan H : *Imaging changes in patients with 2019-nCov.* Vol.30. Springer;2020; pp.3612–3613. 10.1007/s00330-020-06713-z 32025790 PMC7075276

[11] Stogiannos N : COVID-19 in the radiology department: What radiographers need to know [published online ahead of print, 2020 Jun 4]. *Radiography (Lond).* 2020;1078(20):30084–30085. 10.1016/j.radi.2020.05.012 PMC7269964 32532596

[12] Kovács A : The sensitivity and specificity of chest CT in the diagnosis of COVID-19. *Eur. Radiol.* 2020;1–6. 10.1007/s00330-020-07347-x PMC7553375 33051732

[13] Mahmoud H : Can chest CT improve sensitivity of COVID-19 diagnosis in comparison to PCR? A meta-analysis study. *Egypt. J. Otolaryngol.* 2020;36(1):1–7. 10.1186/s43163-020-00039-9

[14] Fu Z : CT features of COVID-19 patients with two consecutive negative RT-PCR tests after treatment. 2020. 10.1038/s41598-020-68509-x PMC7360570 32665633

[15] Xie X : Chest CT for Typical Coronavirus Disease 2019 (COVID-19) Pneumonia: Relationship to Negative RT-PCR Testing. *Radiology.* 2020;296(2):E41–E45. 10.1148/radiol.2020200343 32049601 PMC7233363

[16] Rubin GD : The role of chest imaging in patient management during the COVID-19 pandemic: a multinational consensus statement from the Fleischner Society. *Chest.* 2020;158:106–116. 10.1016/j.chest.2020.04.003 32275978 PMC7138384

[17] Xiong Y : Clinical and high-resolution CT features of the COVID-19 infection: comparison of the initial and follow-up changes. *Investig. Radiol.* 2020;55:332–339. 10.1097/RLI.0000000000000674 32134800 PMC7147282

[18] Wang Y : Temporal changes of CT findings in 90 patients with COVID-19 pneumonia: a longitudinal study. *Radiology.* 2020;296:E55–E64. 10.1148/radiol.2020200843 32191587 PMC7233482

[19] Zheng C : Time course of lung changes at chest CT during recovery from Coronavirus Disease 2019 (COVID-19). *Radiology.* 2020;295:715–721. 10.1148/radiol.2020200370 32053470 PMC7233367

[20] Zhou S : CT features of coronavirus disease 2019 (COVID-19) pneumonia in 62 patients in Wuhan, China. *Am. J. Roentgenol.* 2020;214(6):1287–1294. 10.2214/AJR.20.22975 32134681

[21] Omar S Motawea AM Yasin R : High-resolution CT features of COVID-19 pneumonia in confirmed cases. *Egypt. J. Radiol. Nucl. Med.* 2020;51(1):1–9.

[22] Bai HX : Performance of radiologists in differentiating COVID-19 from viral pneumonia on chest CT. *Radiology.* 2020;200823. 32155105 10.1148/radiol.2020200823 PMC7233414

[23] Ghosh S : Imaging algorithm for COVID-19: A practical approach. *Clin. Imaging.* 2021;72:22–30. 10.1016/j.clinimag.2020.11.022 33197713 PMC7655027

[24] Struyf T : Signs and symptoms to determine if a patient presenting in primary care or hospital outpatient settings has COVID-19. *Cochrane Database Syst. Rev.* 2021;2. 10.1002/14651858.CD013665.pub2 PMC8407425 33620086

[25] Guan W-J : Clinical characteristics of coronavirus disease 2019 in China. *N. Engl. J. Med.* 2020;382(18):1708–1720. 10.1056/NEJMoa2002032 32109013 PMC7092819

[26] Hani C : COVID-19 pneumonia: a review of typical CT findings and differential diagnosis. *Diagn. Interv. Imaging.* 2020;101(5):263–268. 10.1016/j.diii.2020.03.014 32291197 PMC7129663

[27] Mair MD : A systematic review and meta-analysis comparing the diagnostic accuracy of initial RT-PCR and CT scan in suspected COVID-19 patients. *Br. J. Radiol.* 2021;94(1119):20201039. 10.1259/bjr.20201039 33353381 PMC8011239

[28] Chan JF-W : Improved molecular diagnosis of COVID-19 by the novel, highly sensitive and specific COVID-19-RdRp/Hel real-time reverse transcription-PCR assay validated in vitro and with clinical specimens. *J. Clin. Microbiol.* 2020;58(5):e00310–e00320. 10.1128/JCM.00310-20 32132196 PMC7180250

[29] Carotti M : Chest CT features of coronavirus disease 2019 (COVID-19) pneumonia: key points for radiologists. *Radiol. Med.* 2020;125:636–646. 10.1007/s11547-020-01237-4 32500509 PMC7270744

[30] Haytham A : Investigation of the diagnostic importance and accuracy of CT in the chest compared to the RT-PCR test for suspected COVID-19 patients in Jordan.[Dataset]. *Zenodo.* 2023. 10.5281/zenodo.7684523 PMC10562777 37822316

