# Supplementary material for: Investigation of the diagnostic importance and accuracy of CT in the chest compared to the RT-PCR test for suspected COVID-19 patients in Jordan
Source: F1000Res. 2023 Nov 13;12:741. Originally published 2023 Jun 26. [Version 2] doi: 10.12688/f1000research.130388.2 (PMC10562777; doi:10.12688/f1000research.130388.2)
Supplement: Peer review history

# Reviewer report: Reviewer response for version 1

**DOI:** 10.5256/f1000research.143143.r212008

**Published:** 2023-10-09

**Authors:** Ammar A. Oglat

**Affiliations:** 1 The Hashemite University, Zarqa, Jordan

Comments for the authors:

1. The title is comprehensive and written well.   
2. Please note that the conclusion in the abstract section needs to be written in an academic way.
3. Please add further keywords.
4. The introduction part is written in a good academic method. However, please double check the grammatical errors in the introduction paragraphs. 
5. The results and discussion are comprehensive and written well. 
6. The conclusion section should be a concise paragraph explaining further results. 
7. Please check the grammatical and typo errors in the whole article. 
8. Check the values in the whole manuscript.  

Please ensure that the entire manuscript is edited by the authors and someone with proficiency in native English.

 If the required comments are corrected, the manuscript can be accepted for indexing.

 Accepted with minor corrections.

Is the work clearly and accurately presented and does it cite the current literature?

Yes

If applicable, is the statistical analysis and its interpretation appropriate?

Yes

Are all the source data underlying the results available to ensure full reproducibility?

Yes

Is the study design appropriate and is the work technically sound?

Yes

Are the conclusions drawn adequately supported by the results?

Partly

Are sufficient details of methods and analysis provided to allow replication by others?

Yes

Reviewer Expertise:

Medical Imaging Techniques

I confirm that I have read this submission and believe that I have an appropriate level of expertise to confirm that it is of an acceptable scientific standard.

# Response

**Published:** 2023-10-29

**Authors:** haytham alewaidat

**Affiliations:** Jordan University of Science and Technology, Jordan

1. The title is comprehensive and written well.   
2. Please note that the conclusion in the abstract section needs to be written in an academic way.
*Abstract is updated by me.*
3. Please add further keywords.
*Yes, keywords are added.*
4. The introduction part is written in a good academic method. However, please double check the grammatical errors in the introduction paragraphs. 
*All errors and issues are addressed as well as solved.*
5. The results and discussion are comprehensive and written well. 
6. The conclusion section should be a concise paragraph explaining further results. 
7. Please check the grammatical and typo errors in the whole article. 
*All errors and issues are addressed as well as solved.*
8. Check the values in the whole manuscript.  
*Done.*

# Reviewer report: Reviewer response for version 1

**DOI:** 10.5256/f1000research.143143.r204241

**Published:** 2023-10-04

**Authors:** Nitika Panakkal

**Affiliations:** 1 Manipal Academy of Higher Education, Manipal, Karnataka, India

- Typographical errors can be corrected.
- The statistical analysis section needs to be modified to include only the statistical tests used for the study, other details can be added in the introduction/background.
- The relevance of the results of the systematic review can be added in introduction/methods and may be removed from the statistical analysis section.
- Was interobserer agreement done? If so, it can be added which statistical test was done to evaluate that.
- Sensitivity, specificity, PPV and NPV can be added in the statistcal methods used.
- Results report higher specificity in males, tables show higher specificity in females.
- Some of the points in the introduction are repeated in the discussion.
- Some of the limitations of CT can be addressed like dose, cost, time for disinfecting, all which can affect the time required for emergency patients (intro or discussion).

Is the work clearly and accurately presented and does it cite the current literature?

Yes

If applicable, is the statistical analysis and its interpretation appropriate?

Yes

Are all the source data underlying the results available to ensure full reproducibility?

Yes

Is the study design appropriate and is the work technically sound?

Yes

Are the conclusions drawn adequately supported by the results?

Yes

Are sufficient details of methods and analysis provided to allow replication by others?

Yes

Reviewer Expertise:

radiation dose in imaging procedures, computed tomography

I confirm that I have read this submission and believe that I have an appropriate level of expertise to confirm that it is of an acceptable scientific standard.

# Response

**Published:** 2023-10-29

**Authors:** haytham alewaidat

**Affiliations:** Jordan University of Science and Technology, Jordan

- Typographical errors can be corrected.
*All errors and issues are addressed as well as solved.*
- The statistical analysis section needs to be modified to include only the statistical tests used for the study, other details can be added in the introduction/background.
*The statistical analysis section has been updated in the current version.*
- The relevance of the results of the systematic review can be added in introduction/methods and may be removed from the statistical analysis section.
*Yes, the results of the systematic review is inserted in introduction/methods.*
- Was interobserer agreement done? If so, it can be added which statistical test was done to evaluate that.
*Yes, interobserer agreement is added as well as statistical tests such as Chi-square, and Sperman coefficient is also evaluated.*
- Sensitivity, specificity, PPV and NPV can be added in the statistical methods used.
*The table in appendix and its interpretation is added in statistical analysis.*
- Results report higher specificity in males, tables show higher specificity in females.
*I have corrected my results as original results and tables show higher specificity in females.*
- Some of the points in the introduction are repeated in the discussion.
*Repeated data is removed.*
- Some of the limitations of CT can be addressed like dose, cost, time for disinfecting, all which can affect the time required for emergency patients (intro or discussion).
*I have added all limitations and also highlighted them such as dose, cost, time for disinfecting.*
